# Complex mitochondrial DNA rearrangements in individual cells from patients with sporadic inclusion body myositis

**DOI:** 10.1093/nar/gkw382

**Published:** 2016-04-30

**Authors:** Karolina A. Rygiel, Helen A. Tuppen, John P. Grady, Amy Vincent, Emma L. Blakely, Amy K. Reeve, Robert W. Taylor, Martin Picard, James Miller, Doug M. Turnbull

**Affiliations:** 1 Wellcome Trust Centre for Mitochondrial Research, Institute of Neuroscience, Medical School, Newcastle University, Newcastle upon Tyne NE2 4HH, UK; 2 Newcastle University Centre for Ageing and Vitality, Institute of Neuroscience, Medical School, Newcastle University, Newcastle upon Tyne, NE2 4HH, UK; 3 Division of Behavioral Medicine, Department of Psychiatry, Department of Neurology and CTNI, College of Physicians and Surgeons, Columbia University Medical Center, New York, NY 10032, USA; 4 Department of Neurology, Newcastle upon Tyne Hospitals NHS Foundation Trust Royal Victoria Infirmary, Newcastle upon Tyne, NE1 4LP, UK

## Abstract

Mitochondrial DNA (mtDNA) rearrangements are an important cause of mitochondrial disease and age related mitochondrial dysfunction in tissues including brain and skeletal muscle. It is known that different mtDNA deletions accumulate in single cells, but the detailed nature of these rearrangements is still unknown. To evaluate this we used a complementary set of sensitive assays to explore the mtDNA rearrangements in individual cells from patients with sporadic inclusion body myositis, a late-onset inflammatory myopathy with prominent mitochondrial changes. We identified large-scale mtDNA deletions in individual muscle fibres with 20% of cytochrome *c* oxidase-deficient myofibres accumulating two or more mtDNA deletions. The majority of deletions removed only the major arc but ∼10% of all deletions extended into the minor arc removing the origin of light strand replication (O_L_) and a variable number of genes. Some mtDNA molecules contained two deletion sites. Additionally, we found evidence of mitochondrial genome duplications allowing replication and clonal expansion of these complex rearranged molecules. The extended spectrum of mtDNA rearrangements in single cells provides insight into the process of clonal expansion which is fundamental to our understanding of the role of mtDNA mutations in ageing and disease.

## INTRODUCTION

Mitochondrial DNA (mtDNA) rearrangements are a common feature of primary mitochondrial disease due to single, large-scale mtDNA deletions and diseases due to defects in mtDNA maintenance (e.g. POLG, RRM2B) (1,2). MtDNA rearrangements are also an important cellular feature in inflammatory diseases such as sporadic Inclusion Body Myositis (sIBM) and multiple sclerosis (3), certain neurodegenerative disease such as Parkinson's disease and in normal human ageing (4). Despite these observations in-depth molecular characterization and quantitative survey of large numbers of cells with rearranged mtDNA molecules in disease and ageing has been limited (5). Detailed investigation of these rearrangements at a single cell and single molecule level is crucial to elucidate mechanisms of their formation and accumulation in tissues through clonal expansion.

One of the diseases with the most prominent mitochondrial changes is sIBM, a common myopathy of older age, characterized by inflammatory and degenerative features in the affected muscle groups (6). The latter include mitochondrial histopathological abnormalities which manifest as diminished respiratory chain function, abnormal proliferation and subsarcolemmal accumulation of mitochondria (7). Central to sIBM pathogenesis, large-scale mtDNA deletions are considered the major cause of respiratory chain deficiency with a single type of mutation clonally-expanded to detrimental levels in individual myofibres (8–10).

There has been a limited number of studies characterizing mtDNA deletions in sIBM and inflammatory myopathies. Oldfors *et al*. described, for the first time, the presence of mtDNA deletions in biopsies from three sIBM patients using in-situ hybridization detecting *MT-ND2* and *MT-ND4* transcripts (7). Accumulation of mtDNA deletions frequently causes respiratory chain deficiency detected as cytochrome *c* oxidase (COX) enzymatic deficiency. COX-deficient myofibres showed normal levels of *MT-ND2* (minor arc locus) while the majority of them showed reduced hybridization with *MT-ND4* (major arc locus) mRNA, indicative of major arc deletions located between the two origins of replication: Origin of Light and Heavy strand replication, O_L_ and O_H_, respectively. A second study from the same group, using *in situ* hybridization for *MT-ND2, MT-ND4, MT-ND6* and cytochrome b, confirmed in one more patient the presence of major arc deletions, which in some ragged red fibres removed *MT-ND4* and in others *MT-ND6* whilst preserving the remaining genes. Polymerase chain reaction (PCR) analyses of homogenate and single-fibre DNA extracts further demonstrated these major arc deletions of different sizes (7). To date the largest study of deletions in sIBM sequenced 122 deletion breakpoints and characterized 33 different mtDNA deletions from four patients (11). The majority of deleted fragments encompassed only a portion of the major arc of mtDNA (common major arc deletions) but, interestingly, 6 out of the 33 expanded up to the O_L_ removing a part of it, and one removed the entire O_L_ (11).

The major limitation of this and previous studies was that the primers used for PCR-facilitated detection of deletion species of mtDNA were flanking O_L_ region, which only allowed amplification of the major arc deletions. Another group overcame this problem by using a forward primer within the D-Loop region and a reverse primer at position np14570 (12). Investigation of muscle mtDNA from two sIBM and one polymyositis patient revealed multiple deletions, three of which were sequenced. The breakpoints were located at np1734, np1753 and np3326 demonstrating mtDNA deletions spanning far into the minor arc of mitochondrial genome (12). However, this valuable study screening the majority of the mitochondrial genome was limited by the small sample size. In addition, although partial duplications of mitochondrial genome have been described in other conditions with mtDNA deletions (13,14) no studies to date have investigated mtDNA duplications as possible rearrangements in muscle from sIBM patients (5).

In view of the limited knowledge of the molecular defects in mtDNA in sIBM, other inflammatory myopathies and ageing, we identified mtDNA rearrangements in single muscle fibres from patients with sIBM. We used a highly sensitive assay based on whole mtDNA genome PCR amplification from a single mtDNA molecule template (smPCR) and found that multiple mtDNA deletion species coexist in a single myofibre (15). We next applied a newly developed triplex real-time PCR technique allowing detection of three mitochondrial targets which revealed that the majority of these deletions were located within the major arc of the mitochondrial genome but there were also unusual deletions extending into the minor arc. Next generation sequencing of deletion breakpoints confirmed unusually large O_L_-removing molecules and also revealed more complex mtDNA rearrangements containing two deletions in a single molecule. Furthermore, mtDNA duplications were detected in the majority of our patients. This study illustrates an expanded spectrum of mtDNA rearrangements in human skeletal muscle in sIBM that may extend to other inflammatory and neurodegenerative conditions with mitochondrial involvement.

## MATERIALS AND METHODS

### Patients and muscle biopsies

Open muscle biopsies obtained from nine patients with sIBM were analysed in this study. All patients had a diagnosis of clinically-defined or pathologically-defined sIBM according to recent criteria (16). All muscle biopsies (eight from vastus lateralis, one from deltoid) were frozen in liquid nitrogen-cooled isopentane and stored at −80°C until processed. Additionally, DNA extracted from tissue from two patients with single, large-scale mtDNA deletion (muscle), a patient with epilepsy (brain), a patient with Pearson's syndrome (fibroblasts) (two latter DNA samples were kindly provided by Prof Kuntz, Life & Brain Center, Bonn) and five healthy individuals (muscle) were used as controls in different assays. Basic information about the study participants and controls is summarized (Table 1). Consent for research was obtained from all individuals taking part in the study.

**Table 1.** Characteristics of samples used in the study

Case	Patient or Control	Sex	Age at biopsy [years]	Disease duration at biopsy [years]	Tissue sampled
P1	sIBM	M	67	1	Vastus L
P4	sIBM	F	79	10	Vastus L
P5	sIBM	F	64	3	Vastus L
P6	sIBM	F	76	3	Vastus L
P7	sIBM	M	48	4	Vastus L
P8	sIBM	M	81	3	Vastus L
P9	sIBM	F	47	3	Vastus L
P10	sIBM	M	69	3	Vastus L
P11	sIBM	M	52	3	Deltoid
P13	sIBM	M	61	8	Vastus L
s1	single deletion	M	44	16	Vastus L
s2	single deletion	M	38	unknown	Vastus L
c1	epilepsy	F	38	unknown	Brain
c2	Pearson syndrome	F	child	unknown	Fibroblasts
c3	control	F	31	N/A	Hamstring
c4	control	M	47	N/A	Hamstring
c5	control	F	42	N/A	Hamstring
c6	control	F	37	N/A	Hamstring
c7	control	M	40	N/A	Hamstring
c8	control	M	28	N/A	Hamstring

### Histochemical analysis

Cryostat sections (20 μm) were mounted onto superfrost glass slides or PEN membrane slides (Thermo Fisher) and dried for 1h at room temperature. Sequential COX and succinate dehydrogenase (SDH) histochemistry was carried out to detect respiratory-deficient fibres as previously described (17). The stained sections were then used for single-cell molecular analyses.

### Real-time PCR analysis of DNA extracted from single cells

Muscle biopsies from six sIBM patients, frozen as described above, served as a source of individual laser-microdissected myofibres (dissected from 20 μm-thick cryosections, previously labelled for COX/SDH activity, using PALM MicroBeam system, Carl Zeiss) (18). Individual COX-deficient myofibres (*n* = 447) were selected for assessment. DNA was extracted using QIAamp DNA micro Kit (QIagen) according to the manufacturer's instructions. Due to very low concentration of DNA no quantification was undertaken. Detection and quantification of deletion levels were carried out using triplex (*MT-ND4*/*MT-ND1*/D-Loop) real-time PCR assay (TaqMan) (19) (derived from *MT-ND4*/*MT-ND1* duplex assay (20,21)) according to the previously published protocol. Primer and probe sequences are as follows: *MT-ND1* Forward (3485–3504), Reverse (3532–3553), Probe (3506–3529, VIC-labelled MGB probe); *MT-ND4* Forward (12 087–12 109), Reverse (12 170–12 140), Probe (12 111–12 138, FAM-labelled MGB probe); D-Loop Forward (16 536–16 557), Reverse (34-9), Probe (16 559-6, NED-labelled, MGB probe) (primer numbers refer to the mtDNA Genbank reference sequence NC_012920.1). The 25 μl reactions consisted of 1X TaqMan Gene Expression Master Mix, 0.1 μM of each probe and 300 nM of each primer and nuclease-free water. PCR amplification was carried out on StepOnePlus PCR system (Applied Biosystems) using standard cycling conditions as follows: 95°C-20 s, 40 cycles of 95°C-1 s and 60°C-20 s and data were collected using StepOne software v2.1. Data are presented as the amount of either *MT-ND1* or *MT-ND4* in respect to the amount of D-Loop. Due to double/triple-stranded nature of D-Loop region we considered samples normal when *MT-ND1*/D-Loop or *MT-ND4*/D-Loop ratios were between 67% (all mtDNA copies contain triple-stranded D-Loop) and 100% (all mtDNA copies contain double-stranded D-Loop). Real-time PCR result was calculated from a standard curve (seven-point 10-fold dilutions of plasmid p7D1 (19)). Standard curve slopes for all three targets in each experiment never differed by more than 0.05 points and overall the slopes were between −3.46 and −3.32 (indicating PCR amplification efficiency of 94.5 and 100%) with *R*^2^ between 0.9994 and 1. Real-time amplification of single cell-derived DNA would typically provide CT values between 27 and 33, NTCs would either be undetected or amplify at >CT 36. Each sample was assessed in triplicate (limited amount of material did not allow greater repeatability).

### Long range PCR

Long range PCR was carried out using LA Taq DNA polymerase or PrimeSTAR GXL DNA polymerase (TaKaRa, Clontech) on DNA samples extracted from individual myofibres and homogenate muscle samples. For ∼16 kb amplification of single cell-derived DNA several primer pairs located in the proximity of the D-Loop region of mtDNA were used and they are indicated in results Table 4. In case of homogenate DNA samples, F261 (261–285) and R16291 (16 291–16 267) were used. LA Taq reaction (25 μl) was prepared as follows: 1× buffer, 0.4 μM each of dNTPs, 0.4 μM forward and reverse primers, 0.1 unit polymerase and 1 μl DNA extract and nuclease-free water up to 25 μl. Cycling conditions were: 94°C-1 min; 35 cycles of 94°C-30 s, 58°C-30 s and 68°C-16 min; 72°C-19 min. PrimeSTAR GXL reaction (25 μl) was prepared as follows: 1× buffer, 0.2 μM dNTP mix, 0.4 μM forward and reverse primers, 0.625 unit polymerase and 1 μl DNA. Cycling conditions were: 35 cycles of 98°C-15 s and 68°C-12 min. For ∼10 kb amplification PCR F5855 (5855–5875) and R129 (129–110) primers were used. Amplified PCR products were separated through 0.7–0.9% agarose gels. A DNA sample from a patient with no evidence of mtDNA rearrangements was used to ensure full-length amplicons were detected in these assays.

### Single molecule long range PCR (smPCR)

Single molecule PCR enables amplification of mtDNA derived from a single copy of mitochondrial genome (15). Briefly, single muscle fibres were laser dissected from PEN membrane slides using PALM MicroBeam (Carl Zeiss) and collected in 10μl of lysis buffer ‘ATL’ supplemented with Proteinase K according to manufacturer's instructions (QIAGEN QIAamp Micro kit). The tubes were centrifuged at 13 000 *g* for 10 min, incubated at 56°C for 3 h and DNA was extracted using affinity columns according to the protocol. Extracted DNA was then diluted with TE/dH_2_O (1/4 ratio) to such a degree that a PCR product was obtained in less than one out of four reactions. smPCR was carried out in two stages: first round using Phusion Polymerase (Thermo Scientific) and second round using LA Taq (TaKaRa, Clontech). Primer pairs used for the first round were: F2999 (2999–3028) and R2949 (2949–2918) or F2961 (2961–2991) and R2916 (2916–2886) or F130 (130–161) and R16382 (16 382–16 361). PCR products generated with the first pair were used with F3160 and R2494 in the second round of amplification whereas the other pairs were just used again in the second round. Cycling conditions were as described above. Amplicons were separated through 0.5% agarose gels. In all cases the undeleted PCR amplicon (WT) obtained measured around 16 kb.

### Duplication assay

Potential mtDNA duplications or partial duplications were investigated using a protocol described previously (22). Briefly, a PCR was carried out using LA Taq polymerase and the primer pair: 1056F (1056–1075) and 1144R (1144–1123). Cycling conditions were: 95°C-2 min; 10 cycles of 92°C-20 s and 68°C-14 min; 20 cycles of 92°C-25 s and 68°C-16 min; 72°C-10 min. Samples containing only wild-type mtDNA molecules generated an 89 bp product, whereas samples containing duplicated or partially duplicated mtDNA molecules produced a much larger product or multiple products.

### MtDNA deletion breakpoint mapping

In order to map mtDNA deletion breakpoints in smPCR products, a number of PCRs were designed to identify the approximate location of a break point. A range of primers were used to find a pair that would produce a product encompassing the break point (‘stepping-in method’). This product, no larger than 1kb, was then sequenced by conventional Sanger sequencing as described previously (23,24). The 5′ and 3′ breakpoints were defined as the last nucleotide present before the deleted region and the first nucleotide present after the deleted region, respectively. In the situation when the breakpoints were associated with direct repeats the repeat was considered preserved at the 5′ end and removed from the 3′ end.

### MtDNA next generation sequencing

Deep sequencing was used to characterize mtDNA deletion breakpoints in smPCR and long range PCR products. Briefly, selected amplicons, measuring not more than 9 kb, were purified using Agencourt AMPure XP reagent and assessed for concentration and quality using Agilent 12 000 DNA kit on a Bioanalyzer (Agilent 2100, Agilent Technologies). 100ng of each DNA sample were taken for long amplicon libraries preparation using Ion Xpress Plus Fragment Library Kit (Thermo Fisher) according to manufacturer's instructions. The samples were fragmented using Ion Shear Plus Enzyme, then ligated to barcoded adapters. Fragments ∼330 bp in length were recovered with E-gel Size Select 2% gels (Thermo Fisher). The libraries were then amplified and assessed on a Bioanalyzer using the Agilent High Sensitivity DNA Kit. The libraries were pooled in equimolar ratios (final concentration 26 pM), then clonally amplified onto Ion Sphere Particles using the Ion OneTouch system and the Ion OneTouch 200 Template kit v2. Subsequently, the Ion OneTouch ES Instrument allowed enrichment of template-positive Spheres, which were then loaded onto an Ion 314 Chip for deep sequencing on the Ion Torrent system.

For breakpoint mapping of single cell samples, data were analysed using the Torrent Browser (v4.0.2 and above) and alignments to the human mtDNA reference sequence were interpreted using IGV (version 2.3; Broad Institute). The breakpoints were mapped as described in the previous section.

For analysis of homogenate muscle DNA samples, we developed an automated protocol to analyse and extract breakpoints. In brief, reads were aligned to the human mtDNA reference sequence using BWA-MEM (25). Chimeric reads (those aligning to two different parts of the genome on the 5′ and 3′ end of the read) were identified using Bioconductor (26) in R (27) and breakpoints identified. The 5′ breakpoint is reported as the last nucleotide in the mtDNA molecule before the deleted segment, the 3′ breakpoint as the first nucleotide in the mtDNA molecule after the deleted segment. Breakpoint pairs with a read count under 5 were considered not to represent true deletion breakpoints.

## RESULTS

### Different large-scale mtDNA deletions are detected in muscle from sIBM patients

**Figure 1.**
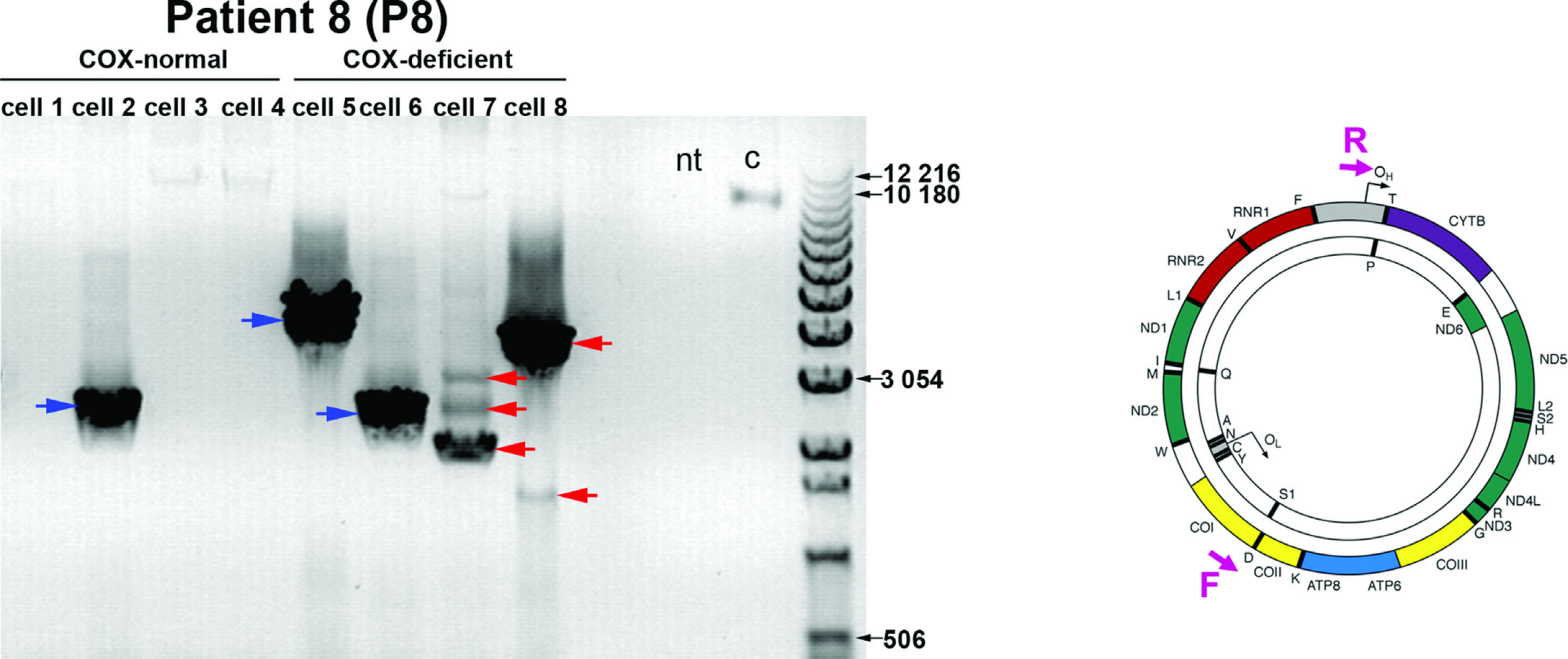
Long range PCR analysis of mtDNA from individual myofibres from a sIBM patient. A representative agarose gel image of 10-kb long range PCR analysis of DNA extracted from four individual COX-normal (‘cell 1–4’) and COX-deficient myofibres (‘cell 5–8’) from a sIBM patient (‘P8’) is presented. A positive control of DNA extracted from whole-blood of a healthy individual (‘c’) was used to ensure detection of full-length amplicons whereas a no-template (‘nt’) control sample served as a control for contamination. A 10-kb product was amplified with wild-type mtDNA (‘c’) whereas shorter products were formed with mtDNA molecules harbouring deletions (‘cell1-4’). A single deletion species was found in ‘cell 1’ and ‘cell 2’ (blue arrows) whilst ‘cell 3’ and ‘cell 4’ contained two or three deletions of different sizes respectively (red arrows). The location of the forward (‘F’) and reverse (‘R’) primers used in the assay (F6358 and R001) is shown in a schematic representation of an mtDNA molecule on the right.

Previously, using quantitative duplex real-time PCR (detecting *MT-ND1* and *MT-ND4* genes), we demonstrated that 55% of COX-deficient myofibres in sIBM patients harboured mtDNA deletions (28). In order to verify and characterize these complex rearrangements, we performed long range PCR analysis of DNA isolated from individual muscle fibres from 9 sIBM patients using primer pairs designed to flank the major arc and amplify approximately 10 kb of wildt-ype mtDNA (Figure 1). Whilst this method is not quantitative, its high sensitivity allows detection of very low mtDNA deletion levels (29). We found that in 78 out of 92 (∼85%) COX-deficient cells mtDNA fragments shorter than the wild type amplicon were generated, confirming the presence of mtDNA deletions in the majority of single biochemically defective cells. The majority of these cells revealed a single amplified product but, interestingly, 12 cells out of the 78 (∼15%) yielded one or two additional amplicons indicating the existence of more than one deletion species in a single cell (Figure 1). Furthermore, sizes of the deleted species varied between individual myofibres obtained from the same muscle biopsy. The majority of COX-positive cells amplified only a wild-type amplicon, but some revealed shorter products (cell 2, Figure 1). This observation demonstrates that low levels of mtDNA deletions are also present in respiratory-normal cells.

### More than one mtDNA deletion accumulates in a single myofibre with a proportion of them being exceptionally large

**Figure 2.**
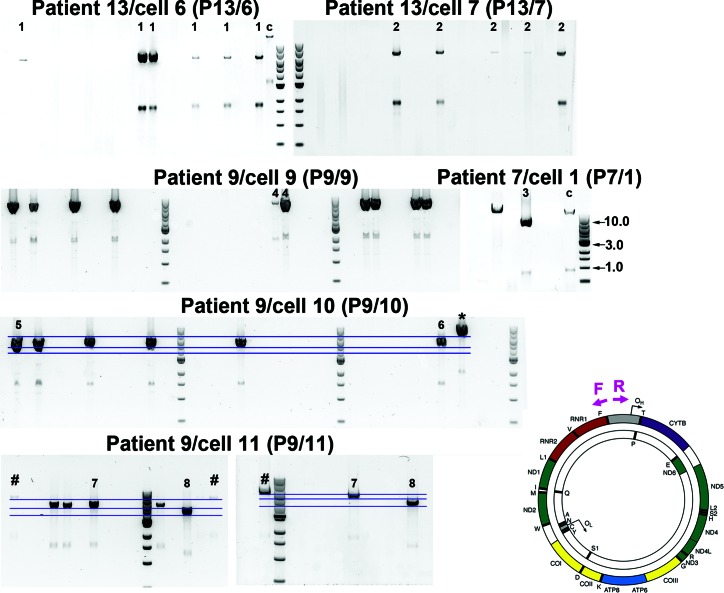
Analysis of individual molecules of mitochondrial genome using single molecule PCR amplification. Representative smPCR gel images from single cell lysates obtained from three sIBM patients (‘P13’, ‘P9’ and ‘P7’), where each amplified product came from a single molecule of mtDNA. In order to ensure that each amplicon was indeed created from one template, the fraction of positives had to be equal or lower than 0.25 (no more than 1 out of 4 PCR reactions contained a product). Primers hybridizing with the D-Loop region were designed to amplify ∼16 kb of mtDNA and are marked as forward (‘F’) and reverse (‘R’) in a schematic of mtDNA molecule on right. One fragment was generated from samples: ‘P13/6’, ‘P13/7’, ‘P9/9’ and ‘P7/1’ signifying one type of mtDNA deletion (additional smaller amplicons, equidistant from the main amplicon in each lane, are a consequence of mispriming). Samples ‘P9/10’ and ‘P9/11’ both gave rise to three amplicons each indicating three different deletion species per cell (blue horizontal lines indicate different sizes of individual fragments). All 20 amplicons marked with numbers (‘1–8’) were sequenced using Sanger sequencing, whereas two products ‘7’ and two products ‘8’ were also verified using next generation deep sequencing. Amplicons ‘*’ and ‘#’ were detected by agarose gel electrophoresis but not sequenced.

To verify whether the multiple short PCR products observed in about 15% of deletion-harbouring COX-deficient myofibres were genuine deletions, we analysed 47 individual, laser microdissected cells from five cases (‘P1’, ‘P7’, ‘P8’, ‘P9’ and ‘P13’) by single molecule PCR (smPCR) (15). We used a variety of primer pairs located within the minor arc, including 2961F and 2916R or 130F and 16382R, which expanded the analysis beyond the major arc. Representative agarose gel electrophoresis images as well as the position of the primers used in the assay are shown in Figure 2. On average, 12 PCR-amplified single molecules were analysed from each cell. We determined that 79% of isolated muscle fibres contained only one type of deletion, a similar result to the one obtained using 10 kb long range PCR described above (85%). We also found that 17% of the analysed muscle fibres contained two deleted products of different sizes, whilst the remaining 4% contained three types of deleted mtDNA species.

**Figure 3.**
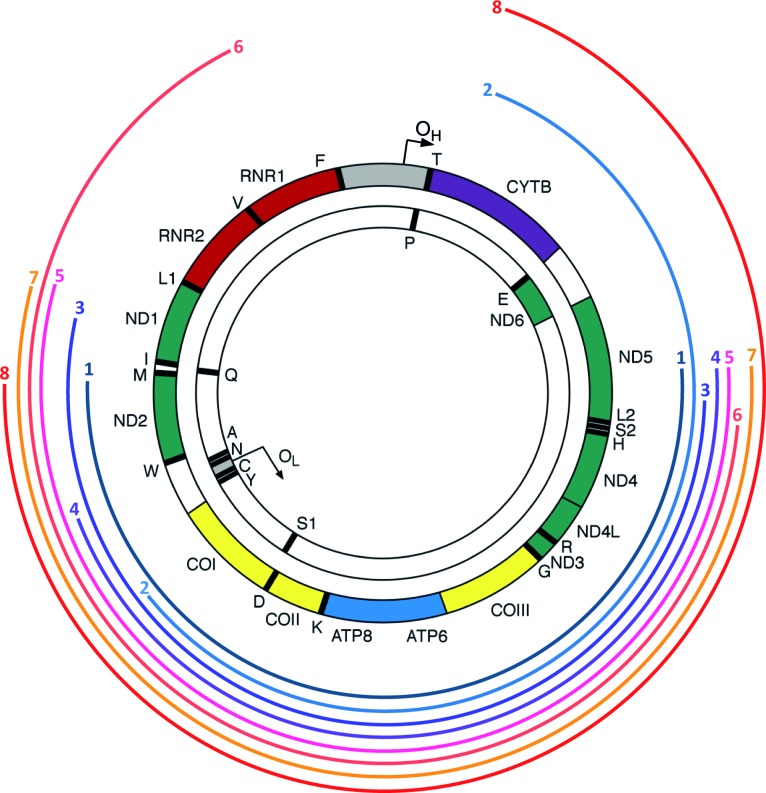
Map of mtDNA deletions. Size and position within the mitochondrial genome of eight types of mtDNA deletions identified by smPCR are presented as coloured curved lines. Line endings on the left and right mark the 5′ and 3′ breakpoints, respectively. Numbers ‘1–8’ correspond to the respective PCR amplicons in Figure 2.

To determine the exact size of the deletion molecules we mapped the deletion breakpoints in 20 PCR products from single molecules (bands marked with ‘1’, ‘2’ etc. in Figure 2) from three patients (‘P7’, ‘P9’ and ‘P13’) by conventional Sanger sequencing. For molecules ‘7’ and ‘8’, we also verified the breakpoints using next generation sequencing. Amongst the 20 PCR products sequenced eight unique mtDNA deletions were found (bands ‘1–8’, Figure 2). The location of each deletion is illustrated in Figure 3. The exact breakpoint positions and sizes of the deletions are specified in Table 2 (nomenclature consistent with Figures 2 and 3). Only one species of clonally-expanded mtDNA deletion was found in single cells from patients 13 and 7:‘P13/6’, ‘P13/7’ ‘P7/1’, marked as ‘1’, ‘2’ and ‘3’ respectively (Figure 2). Three muscle fibres dissected from Patient 9 (‘P9’) demonstrated different scenarios. All of the mtDNA molecules from ‘cell 9’ contained an identical deletion (molecule ‘4’, Figure 2); in contrast, both ‘cell 10’ and ‘cell 11’ contained three different deletion species, two of which were sequenced (‘cell 10’: molecules ‘5’ and ‘6’; ‘cell 11’: molecules ‘7’ and ‘8’, Figure 2) to confirm the existence of multiple mtDNA rearrangements in a single muscle cell (Table 2). Intriguingly, six out of the eight deletions detected removed the origin of light strand replication (O_L_, np5721-5781, http://www.mitomap.org/MITOMAP) (‘1’, ‘3’, ‘5’, ‘6’, ‘7’, ‘8’) and four of them either removed a portion of or the entire *MT-ND1* gene (‘3’, ‘5’, ‘6’, ‘7’, ‘8’). Breakpoints in four of the eight molecules did not occur at sites with repeat sequence; the deletion breakpoints in the four remaining molecules were located in regions with either perfect (*n* = 3) or imperfect (*n* = 1) repeats. Repeated sequences varied from 4 to 11 bp in length (Table 2).

**Table 2.** Characterization of mtDNA deletions found using smPCR assay

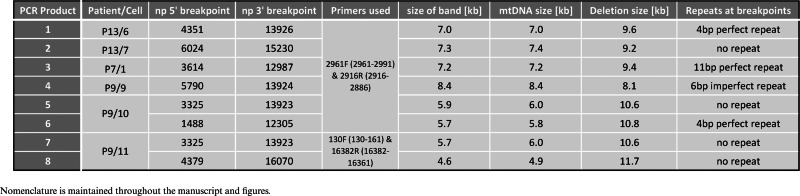								

### Triplex real-time PCR assay allows detection and quantification of the unusual mtDNA deletions

In view of our finding of deletions which remove the *MT-ND1* locus we concluded that the duplex real-time PCR method we initially used (Rygiel *et al*. (28)) is not a suitable tool for comprehensive detection and quantification of human mtDNA deletions. Indeed, the assay can only detect major arc deletions as it relies upon the preservation of *MT-ND1*.This limitation prompted us to design a triplex real-time PCR method, incorporating amplification of three mitochondrial targets: *MT-ND1, MT-ND4* and D-Loop region (Supplementary Figure S1). This assay enables the detection of unusually large mtDNA deletions with 5′ breakpoints located within the minor arc of an mtDNA molecule (19). It calculates the level of either *MT-ND1* or *MT-ND4* relative to the D-Loop region.

Using this method, we analysed a total of 447 laser-microdissected COX-deficient myofibres from muscle biopsies obtained from six sIBM patients (‘P4’, ‘P5’, ‘P7’, ‘P8’, ‘P9’ and ‘P13’, Table 3). We found that 67% of these cells harboured major arc mtDNA deletions, indicated by the loss of *MT-ND4* and preservation of *MT-ND1*. We also detected 4% of cells with isolated *MT-ND1* deletions, where *MT-ND4* was preserved and 4% of cells with deletions encompassing both of these genes (low *MT-ND1*/D-Loop and *MT-ND4*/D-Loop ratios). No deletions were detected in 25% of the assessed myofibres. Individual patients shared a similar trend in the proportion of the three deletion types with the major arc deletions being the most frequent. Two patients (’P9’ and ‘P4’) demonstrated a particularly high level of combined *MT-ND1* and *MT-ND4* deletions (9%) and patient 4 had an exceptionally high (73%) number of cells in which deletions were not detected (Table 3).

**Table 3.** Frequency of different types of mtDNA deletions found in single myofibres from six sIBM patients using triplex real-time PCR analysis

Case	*MT-ND4* deletion		*MT-ND1* deletion		*MT-ND1*&*MT-ND4* deletion		Normal		Total	
	Number of fibres	Percentage of total [%]	Number of fibres	Percentage of total [%]	Number of fibres	Percentage of total [%]	Number of fibres	Percentage of total [%]	Number of fibres	
Patient 4	4	18	0	0	2	9	16	73	22	
Patient 5	44	69	6	9	3	5	11	17	64	
Patient 7	4	67	0	0	0	0	2	33	6	
Patient 8	43	75	1	2	1	2	12	21	57	
Patient 9	58	73	2	3	7	9	13	16	80	
Patient 13	147	67	8	4	4	2	59	27	218	
All	300	67	17	4	17	4	113	25	447	SUM

### Unusual mtDNA deletions in individual myofibres confirmed by deep sequencing

**Figure 4.**
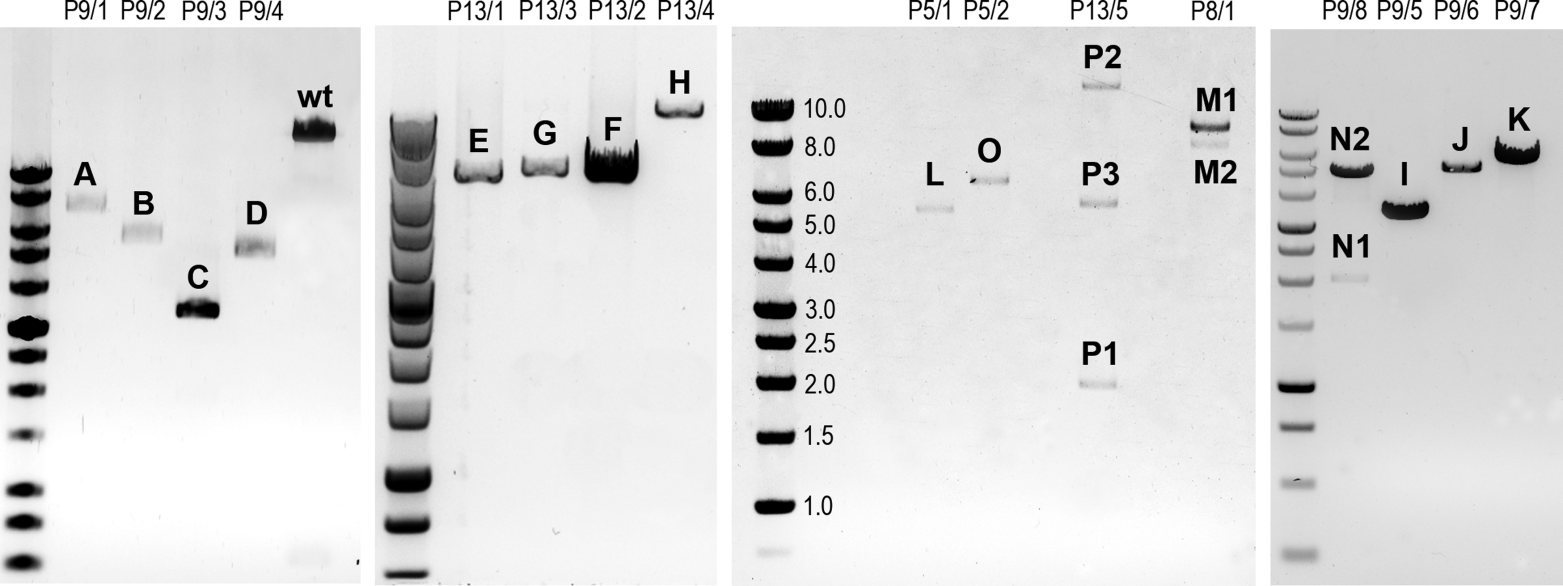
Long range PCR analysis of single myofibre mtDNA. Agarose gel images of 16kb long range PCRs carried out with single cell DNA extracts. The cells were selected based on triplex real-time PCR results; mostly *MT-ND1* and *MT-ND1* & *MT-ND4* deletions were targeted. Each lane is labelled with patient number/cell number (e.g. ‘P9/1’) and each amplified PCR product has a unique identifier: ‘A, B, C’ etc. which is maintained throughout the manuscript. In order to ensure amplification of full-length products with this PCR, whole blood DNA from a healthy individual was used (‘wt’).

**Figure 5.**
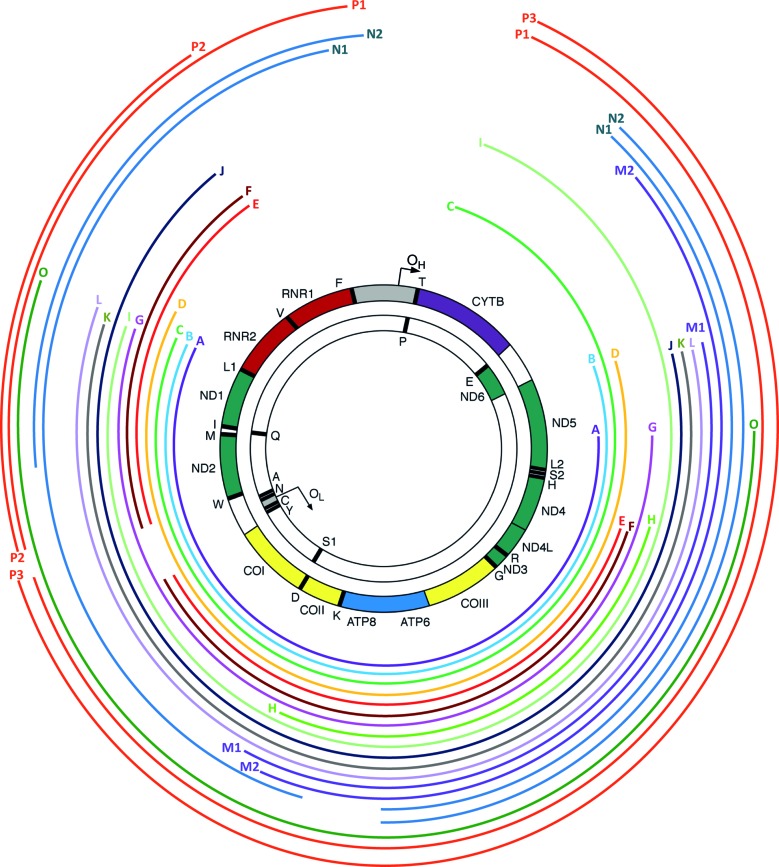
Map of mtDNA deletions. Size and position within the mitochondrial genome of 20 types of mtDNA deletions identified by 16-kb long range PCR are presented as coloured curved lines. Line endings on the left and right mark the 5′ and 3′ breakpoints, respectively. Identifiers ‘A-P3’ correspond to the respective PCR amplicons in Figure 6.

To confirm the triplex real-time PCR results we tested single cell DNA extracts, identified by this assay as containing unusual mtDNA deletions (isolated *MT-ND1* deletion and *MT-ND1* & *MT-ND4* deletion), with whole genome (16 kb) long range PCR and next generation sequencing (Figure 4). The majority of PCRs produced a single, shorter than wild-type, product indicating the existence of only one deletion type per cell. In three out of 16 samples two or three shorter DNA products were amplified (samples ‘P8/1’, ‘P9/8’ and ‘P13/5’, Figure 4). Deletions detected are graphically presented in Figure 5 and detailed information regarding exact breakpoints, primer location and real-time PCR results in Table 4. In the majority of cases the results obtained using triplex real-time PCR were reflected in the breakpoint positions mapped. Samples with both low *MT-ND1*/D-Loop and *MT-ND4*/D-Loop ratios revealed deletions encompassing the major and part of the minor arcs, removing both *MT-ND1* and *MT-ND4* targets. Almost all samples harbouring only one species of these ultra-large mtDNA deletions (‘P9/1-7’ and ‘P5/2’) demonstrated equally decreased ratios of *MT-ND1*/D-Loop and *MT-ND4*/D-Loop ranging from 5 to 70% (Table 4).

**Table 4.** Characterization of mtDNA deletions detected using single-cell long range PCR assay

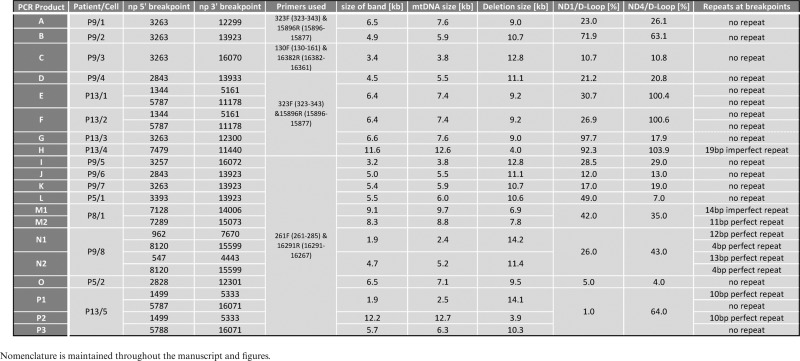										

Myofibres harbouring several rearranged molecules (‘P13/1’, ‘P13/2’, ‘P8/1’, ‘P9/8’ and ‘P13/5’) were also typically in agreement with the quantitative data. In a few instances the agreement between the breakpoints and the triplex assay was not definite, possibly reflecting the observation that multiple deletions can coexist in a single cell. A good example of that is sample ‘P5/1’, where the only deletion detected using long-range PCR encompassed both *MT-ND1* and *MT-ND4*, but real-time PCR analysis revealed *MT-ND1*/D-Loop and *MT-ND4*/D-Loop ratios of 49 and 7%, respectively. It is likely that this discrepancy stands from the presence of another deletion species, with preserved *MT-ND1* and removed *MT-ND4*, which failed to amplify using long range PCR. It should be noted that short major arc deletions are not detectable using the triplex real-time PCR approach. Analysis of molecule ‘H’ from cell ‘P13/4’, in which neither the *MT-ND1* nor the *MT-ND4* primer binding sites were deleted, revealed ∼100% *MT-ND1*/D-Loop and *MT-ND4*/D-Loop ratios (Table 4).

### Mitochondrial genome deletions are more complex than anticipated

Out of the 20 amplicons we characterized, 15 harboured single 5′ and 3′ end breakpoints. In the five remaining amplicons (‘E’, ‘F’, ‘N1’, ‘N2’ and ‘P1’) we identified two 5′ and two 3′ end breakpoints, all contained seemingly within individual molecules rather than two separate, equally sized amplicons, suggesting the occurrence of two deletions in single mtDNA molecules (Figure 4, Table 4). These complex rearrangements, along with the two deletion species identified in sample ‘P8/1’ (Figure 4 and Table 4), were investigated further to establish it they were a real phenomenon or a PCR artefact.

**Figure 6.**
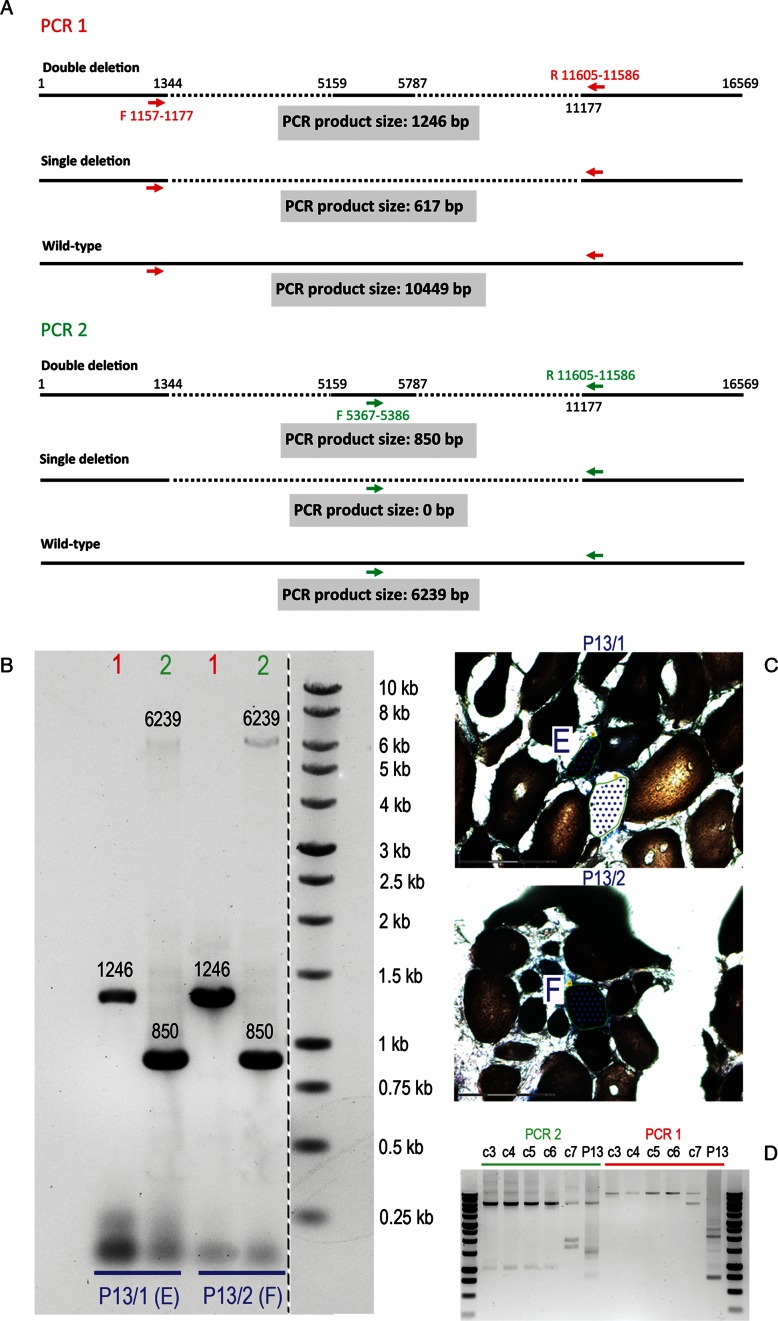
PCR-based validation of deletion breakpoints. Sequencing of 16kb PCR products ‘E’ and ‘F’ derived from two separate cells from the same patient (‘P13/1’ and ‘P13/2’) revealed an identical double-deletion in their mitochondrial genome. In order to confirm this phenomenon, stepping-in PCR was designed. (**A**) ‘PCR 1’ used primers flanking the outer breakpoints of the double-deletion, as indicated by red arrows. Three possible scenarios were considered: (i) the double-deletion was real, in which case the expected product would measure 1246 bp; (ii) there was only a single deletion contained between the outer breakpoints, in which case the product would be 617-bp long; (iii) the PCR would only generate a wild-type amplicon of 10 449 bp. ‘PCR 2’ used a forward primer binding to the short fragment of DNA in between the inner breakpoints and the same reverse primer as used in ‘PCR 1’ (primers indicated by green arrows). Again, three scenarios were investigated: (i) the double-deletion was genuine, in which case the product would measure 850 bp; (ii) a single deletion was real, in which case there would be no amplicon as the binding site for the forward primer would have been removed; (iii) only wild-type would amplify, producing a fragment of 6239 bp. (**B**) Agarose gel showing amplified products from ‘PCR 1’ and ‘PCR 2’ performed on whole-cell lysate from ‘P13/1’ and ‘P13/2’. The amplicons are labelled with the molecular sizes expected given identical double-deletion in both molecules. (**C**) Cells ‘P13/1’ and ‘P13/2’ visualized using COX/SDH histochemistry prior to laser-microdissection for downstream mtDNA analyses. (**D**) Five healthy control muscle homogenate DNA samples (‘c3-7’) and homogenate DNA from the same sIBM patient (‘P13’) were subjected to ‘PCRs 1 and 2’. Control samples ‘c3-6’ produced only wild-type amplicons, whereas control ‘c7’ and ‘P13’ produced additional smaller products indicating presence of deleted species of mtDNA.

Molecules ‘E’ and ‘F’, derived from two COX-deficient myofibres from the same patient (‘P13/1’ and ‘P13/2’, respectively), were found to harbour an identical double deletion. In order to verify this unusual phenomenon, two stepping-in PCRs were carried out on whole-cell DNA extracted from each myofibre. Primers were selected to confirm deletion location within the genome based on the PCR amplicon size (Figure 6A). Elongation time was long enough to facilitate amplification of wild-type molecules. ‘PCR 1’, in which the primers flanked the outer breakpoints, resulted in the amplification of a single product of 1246 bp, consistent with the presence of the two inner breakpoints. ‘PCR 2’, in which the forward primer hybridized with the stretch of DNA between the inner breakpoints, also generated amplicons of the expected sizes for double-deletion (850 bp) and wild-type (6239 bp) mtDNA molecules (Figure 6B). COX/SDH images of ‘P13/1’ and ‘P13/2’ cells prior to laser-microdissection are displayed in Figure 6C. Both PCRs were also performed using five control muscle (‘c1-c7’, characterized in Table 1) and ‘P13’ homogenate DNA samples (Figure 6D). Controls ‘c3-c6’ amplified a single wild-type product in both PCRs, whereas additional shorter amplicons were generated from ‘c7’. The patient's DNA produced multiple products in both PCRs and the wild-type amplicon was entirely missing from ‘PCR 2’ (Figure 6D). These shorter products indicated mtDNA molecules with deletions whose breakpoints were contained between the forward and reverse primers. These shorter amplicons from ‘c7’ and ‘P13’ samples differed in size and abundance (‘P13’ revealed markedly more deletion species than ‘c7’).

**Figure 7.**
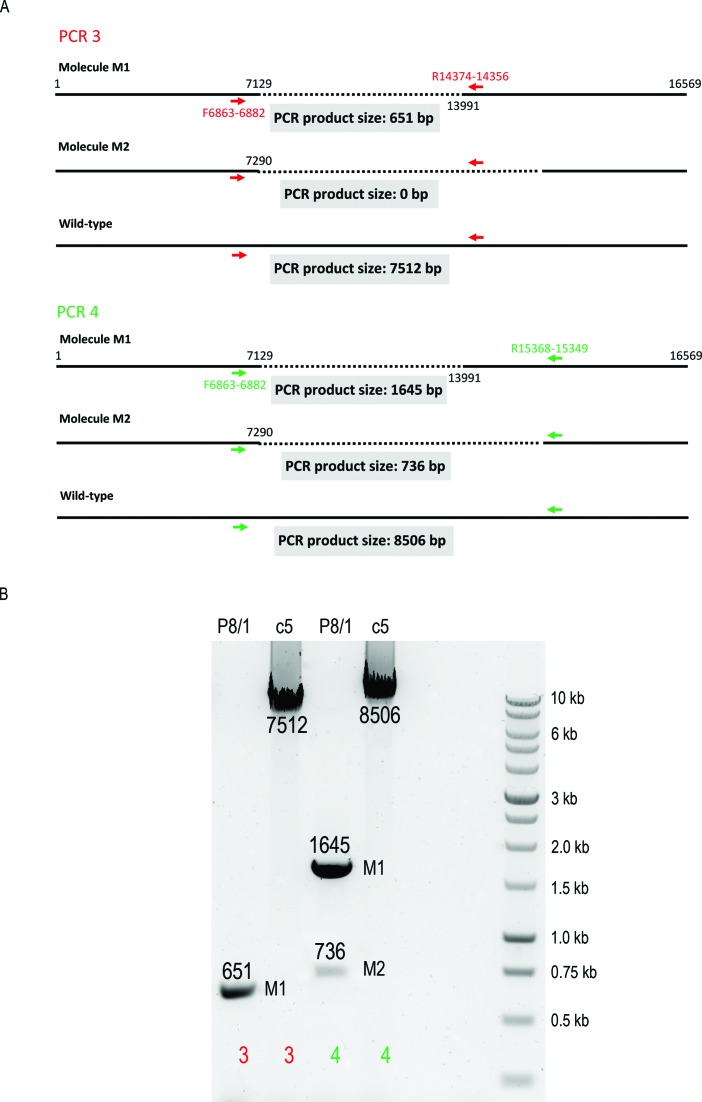
Validation of P8/1 single myofibre long-range PCR result. A total of 16-kb long range PCR produced two products from ‘P8/1’ DNA extract indicating two types of deletion. This was shown by sequencing of the PCR amplicon. In order to validate it further stepping-in PCR method was applied. (**A**) ‘PCR 3’ used primers flanking the shorter deletion (primers indicated by red arrows). Three outcomes were considered: (i) product of 651 bp as a consequence of amplification of molecule ‘M1’; (ii) absence of product should molecule ‘M2’ harbour the only genuine deletion in the sample; (iii) a wild-type product of 7512 bp. ‘PCR 4’ contained primers flanking the larger deletion (green arrows). The possible outcomes were: (i) a single product of 1645 bp if the only deletion molecule present in the cell was ‘M1’; (ii) two products measuring 1645 and 736 bp if both ‘M1’ and ‘M2’ were present; (iii) wild-type band of 8506 bp. (**B**) Agarose gel showing PCR products from ‘reaction 3’ and ‘reaction 4’ carried out using whole-cell lysates from ‘P8/1’ and healthy control homogenate DNA ‘c5’. The amplicons are labelled with appropriate molecular sizes.

A similar PCR approach was applied to the verification of the two deletion species (‘M1’ and ‘M2’) identified in sample ‘P8/1’ (Figure 7A). ‘PCR 3’, with the reverse primer located within the deleted region of molecule ‘M2’, was designed to only detect molecule ‘M1’ (651 bp product), whereas ‘PCR 4’ was designed to amplify both molecules (736 and 1645 bp products) by placing the reverse primer upstream of the 3′ breakpoint of ‘M2’ molecule. Results obtained from both PCRs with ‘P8/1’ single cell DNA were consistent with the existence of two mtDNA deletions (Figure 7B). Wild-type length amplicons of 7512 and 8506 bp were generated from control muscle homogenate DNA ‘c5’ in reactions ‘3’ and ‘4’, respectively. These results confirmed the findings obtained using long range and next-generation sequencing and also proved to be a useful tool in verifying complex DNA rearrangements at a single cell level.

The two remaining samples, ‘P9/8’ and ‘P13/5’, were verified by next-generation sequencing analysis of single-amplicons derived from whole-cell DNA extracts. Breakpoints mapped were identical to the ones obtained previously using a mixed sample containing two (‘N1’ and ‘N2’) or three (‘P1’, ‘P2’, ‘P3’) PCR products per tube (Table 4).

### Characterization of mtDNA deletions at muscle homogenate level

**Figure 8.**
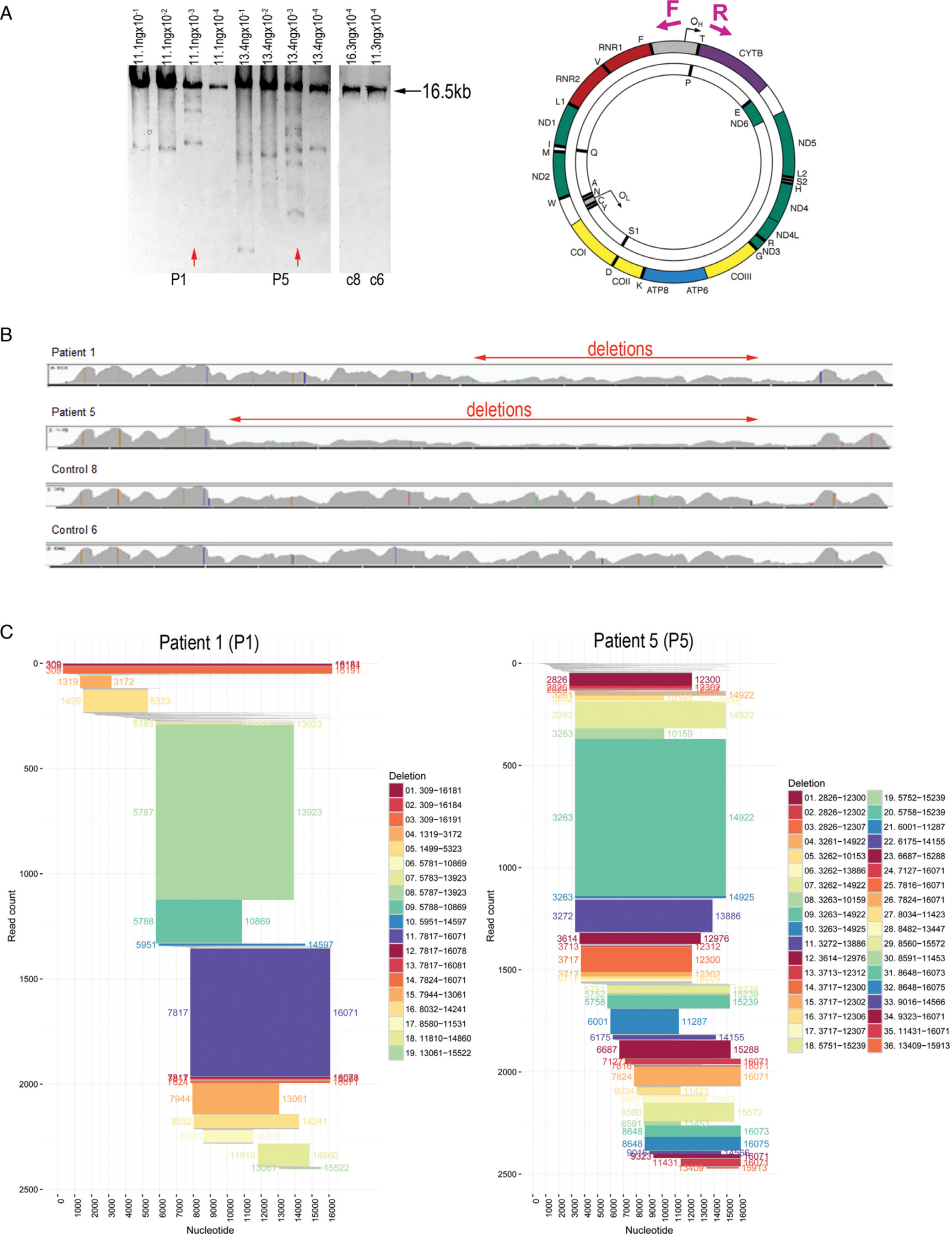
Detection of mtDNA deletions in homogenate DNA samples by next generation sequencing. (**A**) DNA extracted from ten 20-μm muscle cryosections from two sIBM patients (P1 and P5) and two controls (c6 and c8) was subjected to long range PCR. For the patients, four serial dilutions were used in four reactions to find the concentration that would result in the highest number of amplicons. Selected samples are depicted by red arrows. The schematic on the right shows position of PCR primers used (261F and 16291R). (**B**) Graphical representation of read depth for all sequenced samples. Both controls show almost identical peaks and troughs, whereas the patients differ from controls and one another. Low read depth, clearly visible in certain areas of the mitochondrial genome, indicates mtDNA deletions (depicted by red arrows). (**C**) Graphical representation of all deletions detected in the patients’ mtDNA by analysis of chimeric sequencing fragments. Each horizontal bar shows the deleted portion of the mtDNA genome as represented by the chimeric reads that align to two distinct parts of the mtDNA reference sequence. The x axis shows the nucleotide position on the mitochondrial genome (0–16 569 bp). The y axis depicts cumulative read count, with individual reads ordered from top to bottom by 5' and then 3' breakpoints. All chimeric reads are depicted; those in grey have read counts below 5 and are unlikely to represent deletion species. All other multiple read deletions are colour coded for clarity, with breakpoints annotated on both the main figure and in the legend and the height of each bar representing the read count.

Having characterized mtDNA rearrangements in detail using DNA from single cell or single molecule samples we proceeded to assess DNA extracted from muscle homogenates. The homogenate-derived DNA samples from two patients (P1 and P5) and two controls (c6 and c8) were subjected to long range PCR and sequenced using next generation sequencing. Serially diluted templates were amplified with the single cell DNA amplification primer pair (F261 and R16291), which generates ∼16 kb amplicon. Only wild-type amplicons were amplified from the control samples, whereas a range of amplicons of variable sizes were generated from the patients’ samples (Figure 8A). The number of amplicons varied depending on the initial starting concentration of DNA. Samples with the largest number of amplicons were selected for sequencing. The read depth profiles of the control samples were highly similar and markedly different from both patients’ (Figure 8B). The patients’ read depths were clearly diminished in certain areas of the genome, suggesting deletions. It is interesting to note that these areas were not identical for patients 1 and 5. Subsequent analysis of the deletion breakpoints revealed no mtDNA deletions in either of the controls (Supplementary Figure S2) and 19 and 36 deletions in P1 and P5, respectively (Figure 8C and Supplementary Table S1).

### Unusually large mtDNA deletions are accompanied by duplications

**Figure 9.**
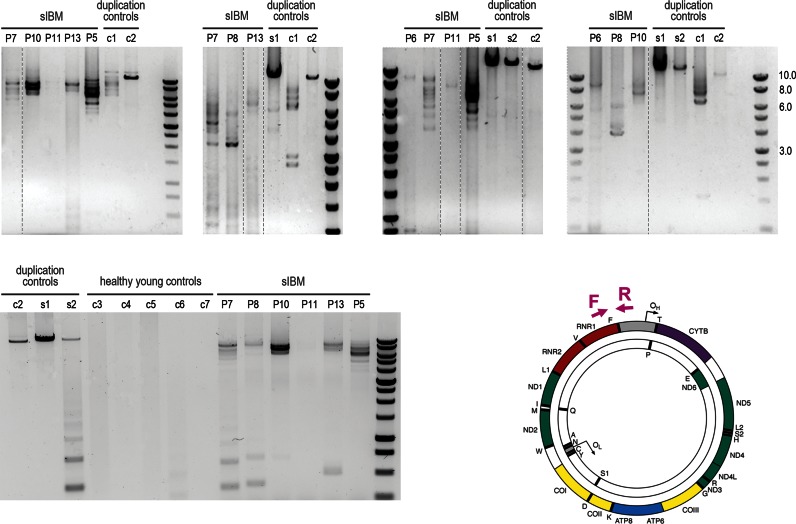
Assessment of duplications of mitochondrial genome. Duplication PCR assay was carried out using 1056F forward (‘F’) and 1144R reverse (‘R’) primers (schematic of mtDNA molecule) on muscle homogenate DNA samples available from seven sIBM patients (‘P5’, ‘P6’, ‘P7’, ‘P8’, ‘P10’, ‘P11’ and ‘P13’). Two control DNA samples known to contain duplications (‘c1’ and ‘c2’) and two samples from patients with single mtDNA deletion (‘s1’ and ‘s2’) were also tested. Agarose gels from several independent experiments are presented. Large products indicate presence of duplicated or partially-duplicated mitochondrial genomes.

The occurrence of mtDNA deletions removing the origin of light stand replication (O_L_) in sIBM muscle fibres is difficult to explain. One of the potential explanations could be that they co-exist with duplicated DNA fragments in which an alternative O_L_ is present allowing for replication and accumulation of these molecules to detectable levels. We tested this hypothesis by applying an established PCR-based methodology (22). This assay relies on a pair of primers strategically located in a region of mtDNA where the majority of duplication breakpoints are expected (schematic of a mitochondrial molecule, Figure 9). If one of more mtDNA duplications are present the PCR generates a high molecular weight product or multiple products. Seven sIBM cases for whom muscle homogenate DNA was available (‘P5’, ‘P6’, ‘P7’, ‘P8’, ‘P10’, ‘P11’ and ‘P13’), two single, large-scale mtDNA deletion mitochondrial patients (‘s1’, ‘s2’) and two positive controls from a patient suffering from epilepsy (‘c1’) and Pearson's syndrome (‘c2’) (kindly provided by Zsurka *et al*. (22)) were tested in multiple independent experiments, some of which are presented in Figure 9. Muscle homogenate DNA samples from sIBM cases repeatedly revealed a pattern of high molecular weight products indicating multiple mtDNA duplications. Single deletion patients’ samples amplified a single product each suggesting that the mtDNA deletion they harbour coexists with a duplicated mtDNA region. Control DNA ‘c1’ and ‘c2’ produced multiple and a single band (as specified by Zsurka *et al*., personal communication), as expected from samples containing either multiple or single rearrangements, respectively.

## DISCUSSION

This study carried out an in-depth and detailed characterization of the molecular defects responsible for respiratory deficiency in individual myofibres from patients with sIBM. Complementary techniques enabled us to identify novel mtDNA rearrangements, expanding the spectrum of possible mtDNA rearrangements in human skeletal muscle. Consistent with a previous study from Oldfors *et al*. we found clonally-expanded, large-scale mtDNA deletions in COX-deficient fibres (7) but, intriguingly, using long range PCR and single molecule PCR, we detected several different mtDNA deletions coexisting in the same muscle fibre in 15 and 20% of respiratory-deficient cells, respectively. Until recently it has been postulated that a single mitochondrial mutation accumulates as a consequence of clonal expansion and, by reaching a threshold level, results in the onset of respiratory deficiency (30). Our data show a different scenario in which there may be expansion of several large-scale mtDNA deletions in a single myofibre, although one deletion may nevertheless predominate. As sIBM is a disease characterized by multiple large-scale mtDNA deletions we hypothesized that these findings would relate to a range of other neuromuscular conditions in which similar mtDNA pathology is present. Indeed we provide evidence that the nature of mtDNA rearrangements we identified in sIBM is not limited to this disease but it occurs in mitochondrial DNA maintenance disease which results in multiple mtDNA deletions accumulating in the muscle (Supplementary Figure S3A). It is likely that other inflammatory and degenerative diseases with mitochondrial involvement share a similar pattern.

It is interesting that at least some forms of hereditary IBM (hIBM) have been shown to present with multiple mtDNA deletions in myofibres (31,32). This finding together with disorders in which multiple mtDNA deletions are a consequence of mutations in nuclear genes engaged in mitochondrial genome maintenance may suggest a nuclear genetic background as underlying mechanism of mtDNA rearrangements formation and accumulation. A number of candidate genes involved in mtDNA replication and maintenance, including *Twinkle, POLG* and *RRM2B*, have been investigated in sIBM patients but the variants found could neither explain the mtDNA rearrangements nor the respiratory deficiency in sIBM (33). Further work involving a broader spectrum of genes is needed to formulate a conclusion whether it is the genetic predisposition, environmental factors or a combination of both that triggers mtDNA rearrangements in this disease.

Further characterization of mtDNA deletions in sIBM at a single cell and homogenate level revealed that in a proportion of fibres the 5′ breakpoint was placed beyond O_L_ and in some cases the deleted region stretched far into the minor arc. Additionally, we detected similar deletions in a patient with mtDNA maintenance disease (Supplementary Figure S3B). This phenomenon of exceptionally large mtDNA deletions, although rare, has been described in literature in other circumstances including physiological ageing. Using *in situ* hybridization, Lee *et al*. reported mtDNA deletions removing O_L_ in 4 out of 26 COX-deficient, ragged-red fibres from senescent monkeys (44). This was later confirmed by PCR analysis and sequencing of DNA from single laser-microdissected cells (45). That study showed that nearly one-third of the genomes had lost O_L_ and a variable portion of the minor arc. Likewise, in healthy human tissue (heart, diaphragm and skeletal muscle) more than half of over 50 clonally-expanded mtDNA deletions removed O_L_ (41). Interestingly, studies involving skeletal muscle from aged rodents did not detect mtDNA molecules devoid of O_L_ (34), potentially suggesting inter-species differences in the mechanisms underlying mtDNA deletion formation. This, however, requires detailed investigation.

Nevertheless, there is scarce evidence supporting prevalence of such O_L_-devoid deletions in human diseases such as sIBM. The only two studies reporting their occurrence are limited by low sample size or the PCR primers used for detection of the deletions (11,12). The four deletion breakpoints these studies characterized: np1741, np1753 (12) and np5561, np5745 (11) were not found in our samples. The closest deletion sites were: np1487 and np1500 as well as np5787 and np5788. We first detected these unusual deletion species by sequencing smPCR products and found that six out of eight molecules lacked O_L_. We concentrated our analyses on smaller products because we suspected they could represent atypical mtDNA rearrangements. As a result, these data do not reflect the true prevalence of these deletions in a population of COX-deficient myofibres. Our triplex real-time PCR-based approach, on the other hand, enabled quantitative assessment of the prevalence of O_L_-eliminating deletions. We tested nearly 500 respiratory-deficient myofibres and demonstrated that approximately 8% of them harboured O_L_-eliminating mtDNA deletions spanning up to at least the *MT-ND1* gene locus (primer binding site). We also note that this figure may be an underestimation of the total number of minor arc deletions as this assay does not detect deletions with a 5′ breakpoint located between O_L_ and *MT-ND1* probe/primer binding site.

Intriguingly, a few of the deletion species we detected contained four breakpoints indicating presence of two deletions in a single molecule. These complex rearrangements of mtDNA were detected in 5 out of 28 molecules subjected to whole mitochondrial genome sequencing. A similar unusual rearrangement of the mitochondrial genome was previously reported by Cao *et al*. in a single ragged red myofibre from a senescent rat (34). The authors identified an mtDNA molecule harbouring several breakpoints and disordered sequence between them. In our study all of the complex molecules harboured two deletions with preserved sequence in between. The majority of deletion molecules detected in this study were different in individual cells but we found two pairs of myofibres containing identical deletion patterns. One pair harboured a single deletion with breakpoints at np3325-13 923 and the other a double deletion (np1344-5161 and np5787-11 178).

Existence of these unusual mtDNA deletions is challenging to explain. O_L_ has been shown essential for mtDNA replication (35) yet a number of molecules lacking O_L_ are detectable in cells. An alternative mtDNA replication model known as strand-coupled replication could take place in myofibres and lead to accumulation of these O_L_-devoid deletions (36). Another way these species could replicate is if they are contained within partially duplicated mtDNA molecules, where O_L_ is preserved within the duplicated region. MtDNA duplications have been reported in a number of disorders including single mtDNA deletions (13), diabetes mellitus (37), Kearns-Sayre syndrome (14), Pearson syndrome (38,39) and adult-onset progressive myopathy (40). Collectively, duplications have been found in over 50% of single mtDNA deletion conditions to date (5). Interestingly, duplications have also been found in skeletal muscle from healthy older adults. PCR analysis of O_L_-lacking clonal mtDNA molecules revealed that these were in fact partially duplicated molecules, with a deleted genome incorporated in a wild-type molecule (41), although the mechanisms underlying such rearrangements remain unclear. Tandem duplication and triplication of an mtDNA segment was also reported in the muscle of a healthy elderly man (42). In this study we demonstrate the existence of duplicated mitochondrial genomes in muscle from sIBM patients. All of the tested sIBM cases harboured multiple duplicated fragments in muscle homogenates. As a control, we used samples from three patients harbouring single, large-scale mtDNA deletions, which all demonstrated a single duplicated mtDNA product as expected, and a sample from a previously characterized patient with epilepsy which contained multiple duplications. We did not detect any duplications in five young healthy control muscle samples.

A strikingly similar pattern of deletion/duplication breakpoints was reported in muscle from patients with *MGME1* mutation (22), with the difference that the absolute majority of rearrangements found there were within the minor arc. In both sIBM and MGME1, distinct breakpoint hotspots exist around tRNA^Leu^ gene (np3230–np3304) at the 5′ end and position np16070 at the 3′ end. In sIBM the 5′ breakpoint of nine out of 28 molecules clustered between np3263 and np3393 and five had ∼np16070 breakpoint but there were also two additional hotspots: ∼np13923 (nine molecules) and ∼np12300 (four molecules). It is interesting to note that the region np16 001–16 100 is a hotspot for breakpoints in patients with multiple mtDNA deletions, sIBM and healthy ageing in contrast to patients with single deletions including Progressive External Ophthalmoplegia (PEO), Kearns-Sayre Syndrome (KSS) and Pearson Syndrome (PS), who do not harbour deletion breakpoints in that region (5). A case study on skeletal muscle from a healthy 57 year old man demonstrated partial triplication of mtDNA with deletion breakpoints located at np3263-3272 and np16 065–16 076 (42). Non-pathogenic mtDNA deletions called sublimons characterized in healthy, mostly cardiac, tissue contained a similar breakpoint pattern: ∼np3260 and ∼np16070 (43). Deletions identified in two homogenate DNA patient samples by next generation sequencing revealed similar hotspots; some present in both and others only in one of them. Although this PCR amplification-based method is unsuitable for quantifying abundance of different deletions in a sample, it identifies a wide range of them. The 5′ breakpoints located at np3200-3300 were common in P5 and absent in P1, whereas breakpoints at ∼np5780 predominated in P1 and were not detected in P5. Similarly, 3′ breakpoints at ∼np13923 were identified only in P1 and ∼np12300 only in P5. The breakpoints common to both patients were located around np5780 and np16 070.

Analysis of DNA sequences flanking the breakpoints in this study provided variable results. Some breakpoints were associated with perfect repeats measuring from four to 13bp, some had imperfect repeats up to 19 bp but the majority did not have any repeated sequences. It is difficult to stipulate a mechanism underpinning deletion/duplication formation in sIBM at this stage and further investigation needs to be carried out to address that.

This study has demonstrated that mtDNA rearrangements are substantially more complex than previously assumed. We used a single-cell approach with a combination of real-time PCR, long range PCR, single molecule PCR and whole mitochondrial genome sequencing to investigate a large number of samples from nine sIBM patients. Overall, our data provide strong evidence for the existence of multiple deletion species in single respiratory-deficient myofibres, as well as complex rearrangements resulting in mtDNA molecules which (i) lack O_L_, (ii) harbour double-deletions and (iii) contain partial duplications. We also quantified the prevalence of different deletion types in a large population of COX-deficient muscle fibres, providing estimates of their abundance. These novel findings thus unearth an expanded spectrum of mtDNA rearrangements in sIBM, which could extend to other inflammatory and degenerative conditions with known mitochondrial involvement and in nuclear mutation-associated mtDNA maintenance disorders and ageing itself. Our data obtained from three cases of nuclear mutation-associated mtDNA disorders demonstrate presence of multiple mtDNA deletions in individual myofibres, with some of them exceptionally large, O_L_-removing and strikingly similar to the ones we have described in sIBM (Supplementary Figure S3). More work carried out on specific cohorts of individuals affected with inflammatory, metabolic and defined genetic conditions is required to reveal the mechanisms by which these complex rearrangements occur, the process driving their accumulation in individual cells and their pathophysiological significance.

## Supplementary Material

Supplementary Data

SUPPLEMENTARY DATA
