# Supplementary material for: Complex mitochondrial DNA rearrangements in individual cells from patients with sporadic inclusion body myositis
Source: Nucleic Acids Res. 2016 Apr 30;44(11):5313–29. doi: 10.1093/nar/gkw382 (PMC4914118; doi:10.1093/nar/gkw382)
Supplement: Supplementary Data [file gkw382_Supplementary_Data.zip › nar-00395-h-2016-File014.pdf]

Supplementary Figure 1

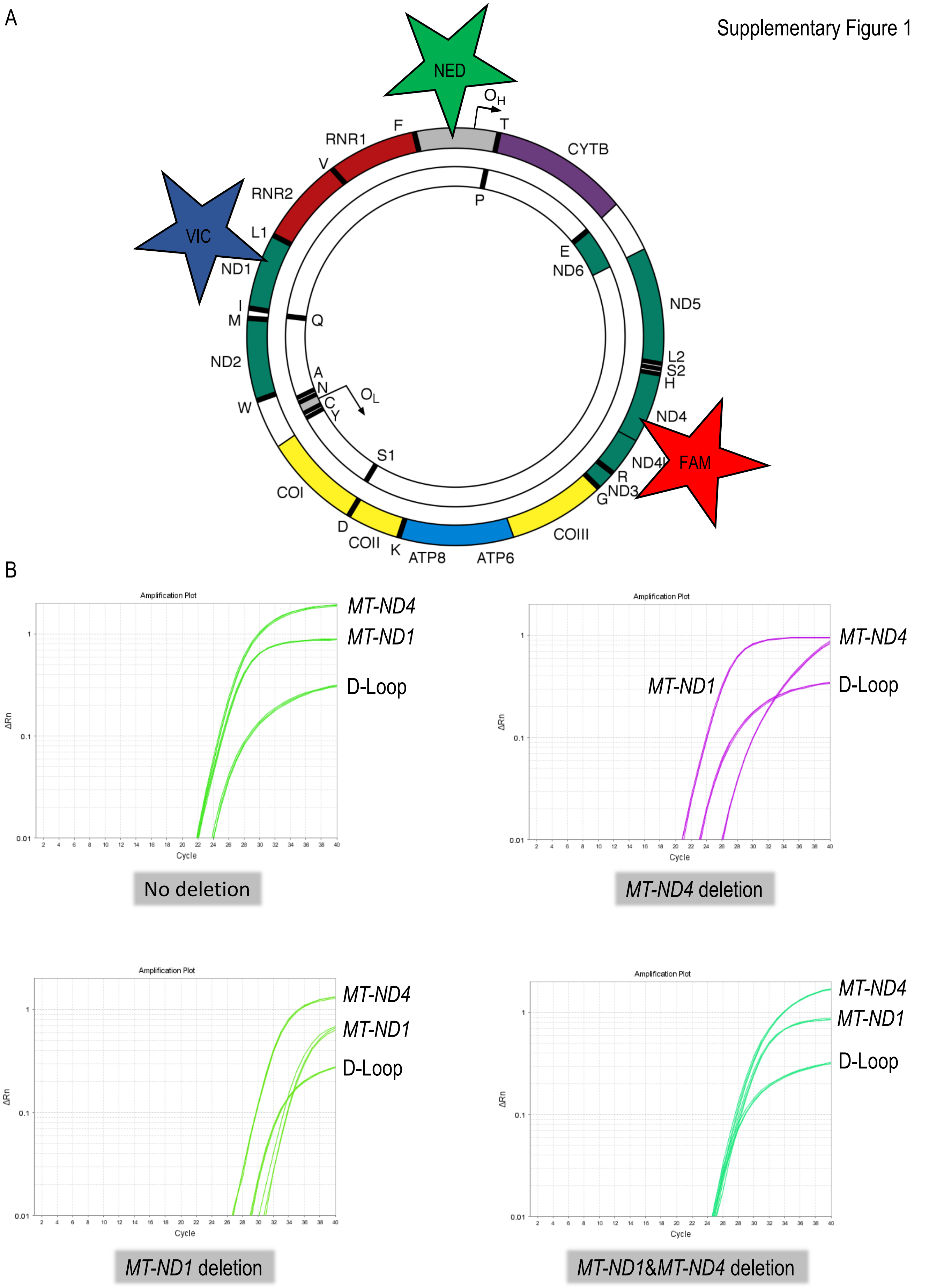

Supplementary Figure 1. Triplex real-time PCR assay developed as a tool to detect and quantify majority of mtDNA deletions in single cells. (A) It allows simultaneous detection of three mitochondrial targets: *MT-ND1*, *MT-ND4* and a region of D-Loop using TaqMan probes labelled with VIC, FAM and NED respectively. Inclusion of D-Loop target enables identification of unusual deletions extending into mitochondrial minor arc. (B) Amplification curves from representative samples containing: wild-type mtDNA (“No deletion”), major arc deletion with *MT-ND4* removed and *MT-ND1* preserved (“*MT-ND4* deletion”), minor and/or major arc deletions with *MT-ND1* removed and *MT-ND4* preserved (“*MT-ND1* deletion”) and minor and major arc deletion with both *MT-ND1* and *MT-ND4* removed (“*MT-ND4* & *MT-ND4* deletion”).
