# Supplementary figures and images for: Complex mitochondrial DNA rearrangements in individual cells from patients with sporadic inclusion body myositis
Source: Nucleic Acids Res. 2016 Apr 30;44(11):5313–29. doi: 10.1093/nar/gkw382 (PMC4914118; doi:10.1093/nar/gkw382)

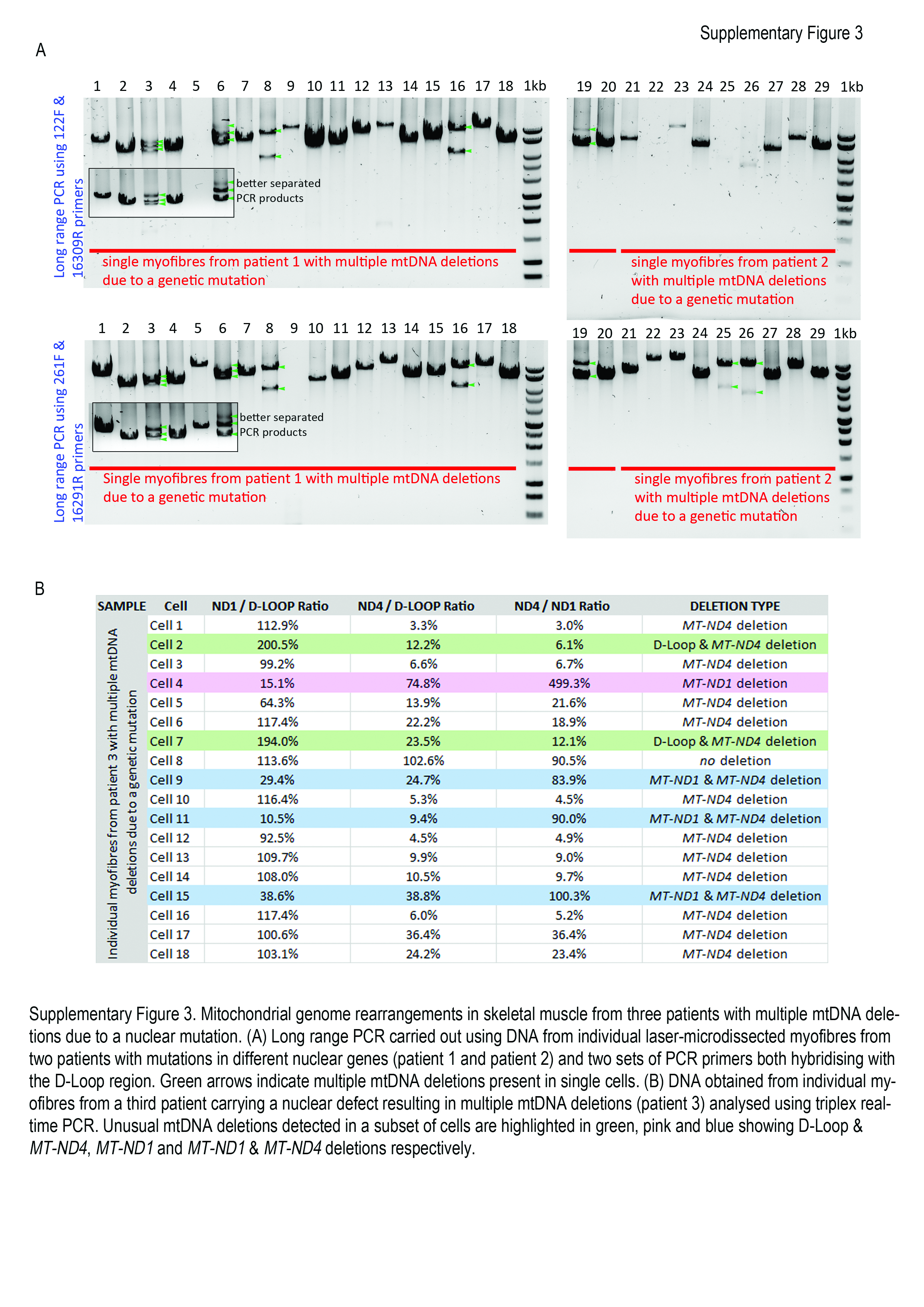

Supplement: Supplementary Data [file gkw382_Supplementary_Data.zip › nar-00395-h-2016-File016.tif]
