# Supplementary material for: Complex mitochondrial DNA rearrangements in individual cells from patients with sporadic inclusion body myositis
Source: Nucleic Acids Res. 2016 Apr 30;44(11):5313–29. doi: 10.1093/nar/gkw382 (PMC4914118; doi:10.1093/nar/gkw382)
Supplement: SUPPLEMENTARY DATA [file supp_44_11_5313__index.html]

Complex mitochondrial DNA rearrangements in individual cells from patients with sporadic inclusion body myositis — Complex mitochondrial DNA rearrangements in individual cells from patients with sporadic inclusion body myositis — SUPPLEMENTARY DATA 

# Complex mitochondrial DNA rearrangements in individual cells from patients with sporadic inclusion body myositis

## SUPPLEMENTARY DATA

- SUPPLEMENTARY DATA
- SUPPLEMENTARY DATA
- SUPPLEMENTARY DATA
- SUPPLEMENTARY DATA
